# SG-APSIC1076: Bacteremia caused by *Streptococcus mitis* in a hematology unit

**DOI:** 10.1017/ash.2023.86

**Published:** 2023-03-16

**Authors:** Ismail Bin Sazali, Lee Lai Chee, May Kyawt Aung, Molly How Kue Bien, Tan Kwee Yuen, Hatijah Binti Tohid, Ling Moi Lin

**Affiliations:** Singapore General Hospital, Singapore; Singapore General Hospital, Singapore; Singapore General Hospital, Singapore; Singapore General Hospital, Singapore; Singapore General Hospital, Singapore; Singapore General Hospital, Singapore; Singapore General Hospital, Singapore

## Abstract

**Objectives:** *Streptococcus mitis* is a gram-positive coccus and is a common commensal found in the throat, nasopharynx, and mouth. In an immunocompromised host, *S. mitis* opportunistically multiplies and can translocate to other sites. At baseline, the prevalence of *S. mitis* remained stable among hematological patients, averaging ~1 case monthly. However, in August–September 2020, 5 *S. mitis* cases were documented in a hematology ward and included overlapping inpatient stays. In this descriptive cluster report, we sought to identify the reasons for the increased prevalence of *S. mitis* in our institution. **Methods:** A literature review was undertaken to gain a better understanding of the bacteriology of *S. mitis*. Subsequently, geographical mapping was performed to identify epidemiological links. Further culture and sensitivity testing was requested. Hand hygiene compliance, environmental audit, and handling of central lines within the ward were examined for any lapses in practice. **Results:** Based on geographical mapping, no epidemiological linkages were established between patients; they were admitted to different rooms and did not share any equipment. Moreover, based on the antibiogram, different bacteria sensitivities were recorded across the isolates from these patients. A hand hygiene and environmental audit result showed 100% compliance. Nurses performed care of central lines in accordance with guidelines. However, an investigation of changes in practice revealed that the use of a toothbrush had only recently been permitted as part of streamlining oral care for hematology patients. Because toothbrushes were not provided by the hospital, patients were utilizing their personal toothbrushes with no direct supervision of their oral care regimen. **Conclusions:** The prevalence of *S. mitis* in hematological patients was likely due to the neutropenic condition of patients. This report provides valuable information supporting the optimization of oral hygiene in immunocompromised patients while minimizing the risk of opportunistic infections.

